# Diffraction Imaging (Topography) with Monochromatic Synchrotron Radiation

**DOI:** 10.6028/jres.093.151

**Published:** 1988-10-01

**Authors:** Bruce Steiner, Masao Kuriyama, Ronald C. Dobbyn, Uri Laor

**Affiliations:** National Bureau of Standards Gaithersburg, MD 20899

**Keywords:** crystal growth, electrooptic materials, monochromatic diffraction imaging, synchrotron topography

## Abstract

Structural information of special interest to crystal growers and device physicists is now available from high resolution monochromatic synchrotron diffraction imaging (topography). In this review, the importance of superior resolution in momentum transfer and in space is described, and illustrations are taken from a variety of crystals: gallium arsenide, cadmium telluride, mercuric iodide, bismuth silicon oxide, and lithium niobate. The identification and detailed understanding of local variations in crystal growth processes are shown. Finally, new experimental opportunities now available for exploitation are indicated.

## 1. Sources of Current Interest

New types of materials with enormous scientific and practical interest are now being created by atomic or “ultramolecular engineering” [1] of structures not found in nature. Successful fabrication of such materials, whether novel in structure or simply far freer of imperfections than found in nature, depends on detailed structural information. The degree of perfection that has been achieved, the effects of remaining defects, and sources of deviation from the intended structure all constitute the type of information required. More fundamental guidance that will underlie future advances in materials design concepts will come from knowledge and understanding of modification in properties engendered by specific variations in structure.

## 2. Types of Information Now Available through Diffraction Imaging

**Figure 1:**
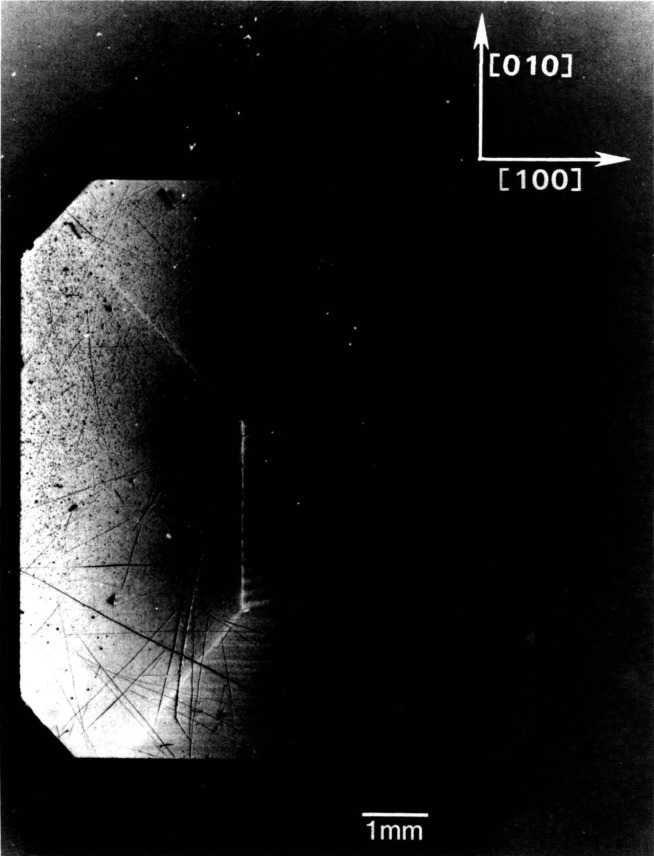
Strain patterns in the 8 keV (309) diffraction from a (001)-cut crystal (slice K) of bismuth silicon oxide.

**Figure 2:**
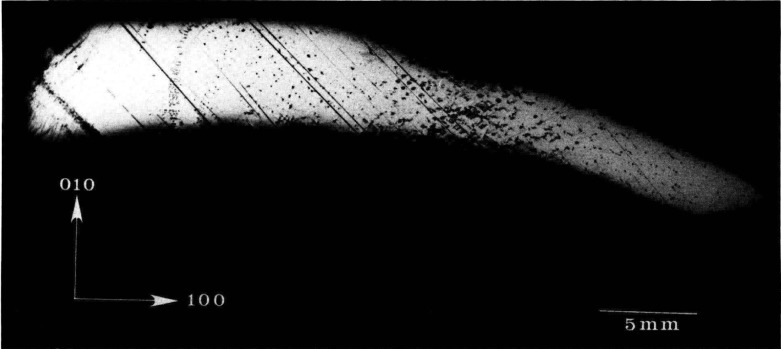
Dislocation patterns in the 10 keV (040) diffraction from an indium doped (001)-cut wafer of gallium arsenide.

In contrast to electron microscopy, which provides information on the *location* of features in *small* regions of materials, diffraction imaging can portray minute *deviations* from crystal perfection over *larger* areas. Diffraction imaging by monochromatic synchrotron x-radiation [2] pinpoints and permits analysis of irregularities that can affect physical properties of materials. For example, lattice strains lead to strong contrast in such images, as in figure 1; and analysis of the patterns in these strains can lead to a detailed understanding of how they arise [3]. Similarly, crystallographic dislocations stand out in the images of high quality crystals, as seen in figure 2.

Analysis of such images can provide not only evidence of the presence and distribution of structural inhomogeneities but also an understanding of their structure and origins. As a result, steps can be taken to enhance or reduce their presence, as desired. Diffraction imaging thus provides useful information on variation in high quality crystals that can be correlated with specific aspects of their performance. Such a correlation can lead to improvement in device performance through guidance on desirable modification in crystal growth and subsequent processing parameters.

## 3. Current Experimental Opportunities

**Figure 3:**
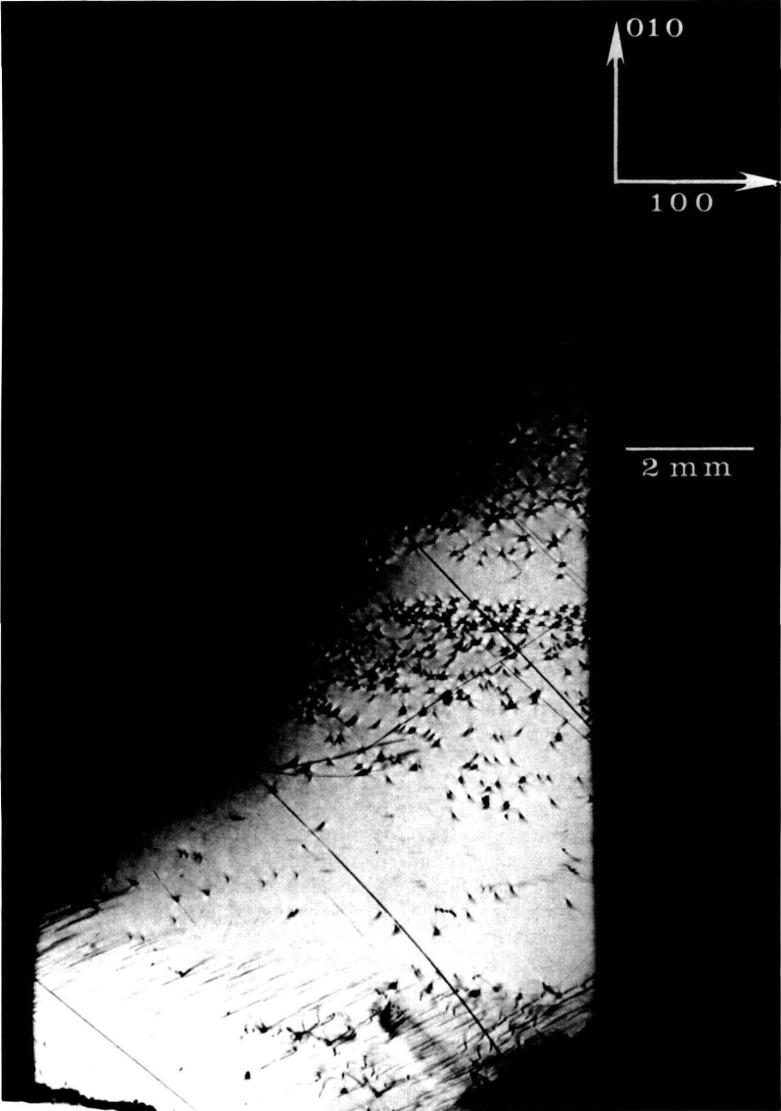
Individual dislocations resolved in 8 keV (400) diffraction from the indium doped (001)-cut wafer of gallium arsenide shown in the previous figure, after fracture.

Synchrotron storage rings now permit far more comprehensive and useful realization of these opportunities than can be achieved with laboratory sources because of three optical characteristics of such rings: their effective small source size, their high brightness, and the presence of a continuous energy spectrum. The small optical source size permits formation of highly parallel x-ray beams, which are required for maximum resolution. The smallest source size achieved to date, 140 micrometers, has been realized in the large storage ring at the National Synchrotron Light Source at Brookhaven National Laboratory. Optics at the end of one of its 20 meter beamlines can achieve at least 1.5 arc-second angular resolution, or less than a micrometer on the surface of a detector in a typical configuration. This resolution can be further increased through suitable optics. With the optics described here [4–6], individual dislocations are clearly seen as in figure 3.

**Figure 4:**
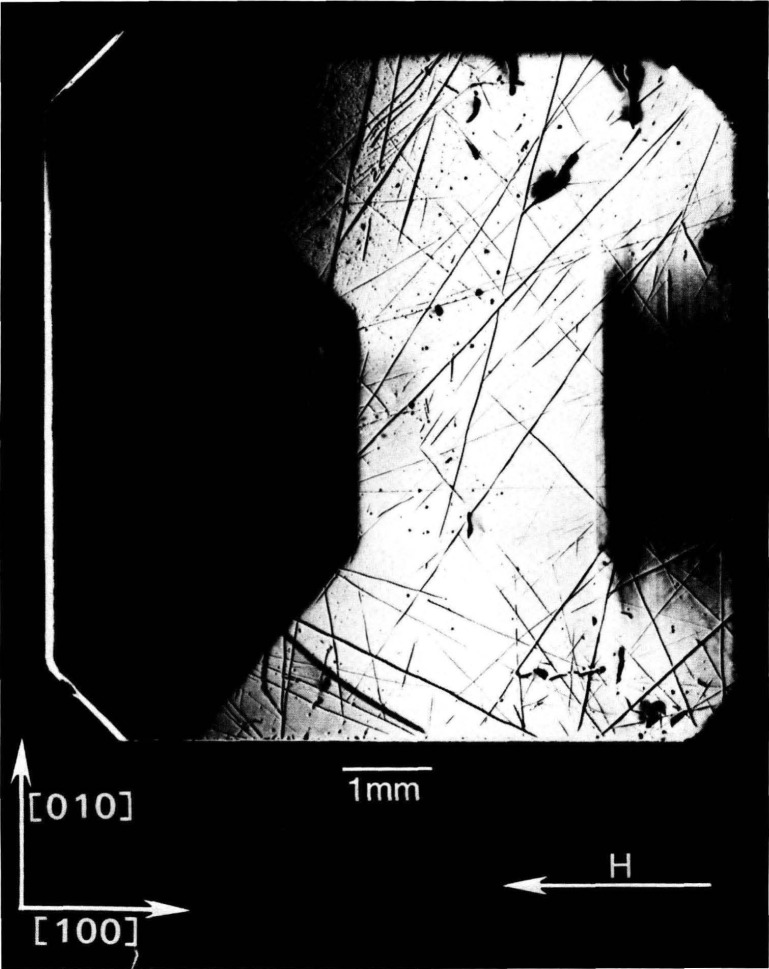
Image of 13.4 keV diffraction in Laue geometry (transmission) from ( $\bar{6}00$) planes of the (001)-cut bismuth silicon oxide crystal shown in figure 1 in diffraction in Bragg geometry.

The high brightness achieved in storage rings is central to several aspects of crystal characterization. First, high brightness enables one to insert additional optics for the improvement of angular and spatial resolution while maintaining a useful flux of photons, as described later. Second, high brightness permits observation of diffraction features in transmission. The distinctive signatures of the dislocations observed in figure 3 are visible only in diffraction in Laue geometry (transmission), with its integration of information from the interior of a crystal. Moreover, when strains are observed in transmission, as in figure 4, differences in their orientation in various parts of the image can be emphasized. In figure 4, for example, strains in the <100> direction are clearly visible in some parts of the crystal while absent in others that appear equivalent in diffraction in Bragg geometry. Analysis of such differences provides information on the processes leading to their formation [3]. Third, the high brightness of a storage ring permits video camera observation of diffraction images in real time. This in turn facilitates surveys of subgrain boundary structure and of large scale crystal strain that are difficult to observe and prohibitive in time by any other means. Also important is the capability for *in situ* experiments under environmental variation of conditions such as temperature, pressure, or electric and magnetic fields.

**Figure 5:**
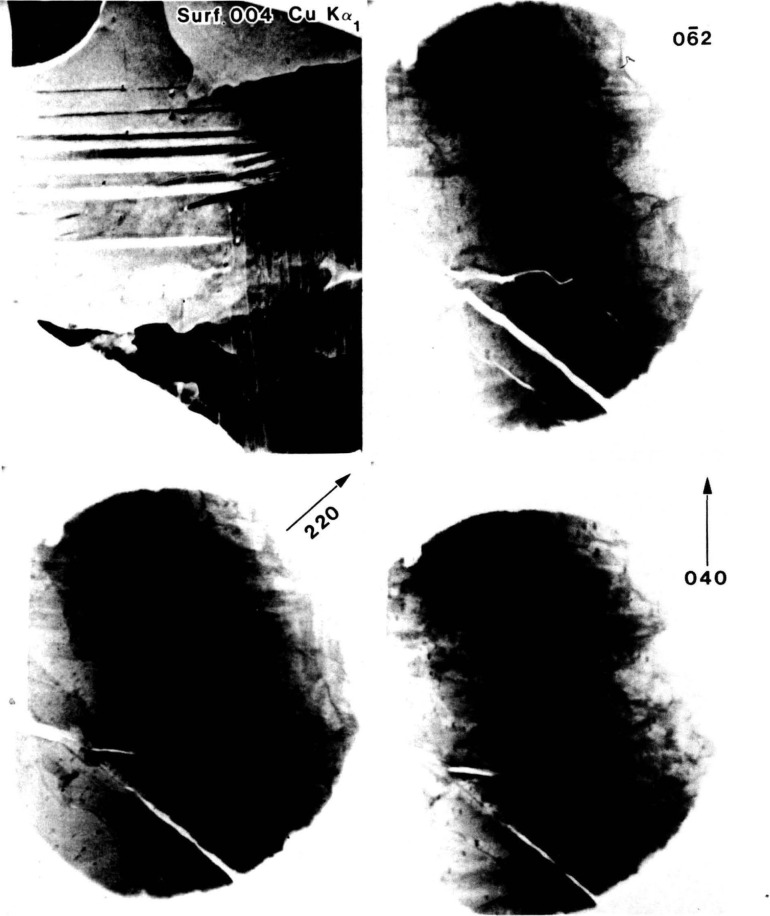
Comparison of a monochromatic laboratory (004) diffraction image of iron-aluminum crystal with three white beam synchrotron topographs: ( $0\bar{6}2$), (220), and (040) of the same crystal.

The availability of a choice in wavelength permits work in transmission that would otherwise not be possible because of absorption. The utilization of a monochromator with the continuum from a synchrotron storage ring can provide important further advantages. The restriction of diffracting radiation to a narrow spectral band substantially increases the contrast in an image over that obtainable with a white beam as in figure 5. The interpretation of such monochromatic images is far simpler and more certain than the interpretation of images formed with white radiation.

When asymmetric diffraction is employed in the monochromator, two additional benefits can be obtained [8]. First, the beam can be expanded several fold to a height of several centimeters. With such a beam, large crystals can be examined without serious interferences from spurious contrast generated by scanning. Second, the parallelism of the analyzed beam is increased by the same factor, bringing the resolution well under one arc-second. This can be reduced to much less than a tenth of an arc-second with appropriate monochromator optics where necessary.

**Figure 6:**
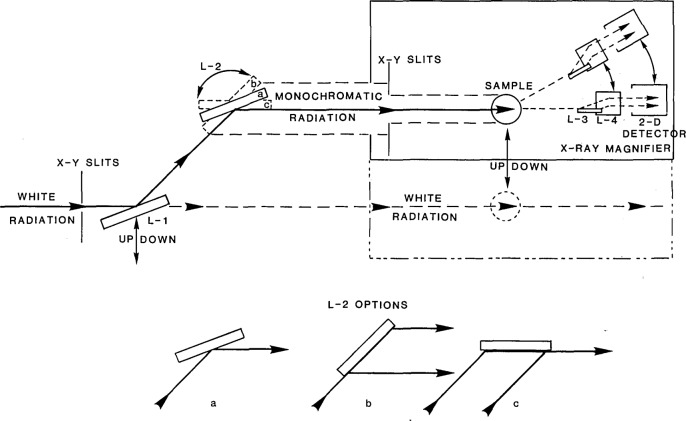
NBS materials science beamline optics: arrangement of flat crystal diffraction elements used for imaging. L1: first monochromator crystal, L2: second monochromator crystal, L3: first crystal of x-ray magnifier, and L4: second crystal of x-ray magnifier.

These advantages are being realized on the National Bureau of Standards (NBS) Materials Beamline X23A-3 at the National Synchrotron Light Source (NSLS) at the Brookhaven National Laboratory [5]. Figure 6 shows the x-ray optical configuration for diffraction imaging at this beamline [6,7]. Two flat crystals (L1 and L2) in sequence control beam divergence and beam size and are arranged to keep the beam position with respect to the sample location fixed in space, independent of the energy selected for study. The first monochromator crystal (L1) intercepts the white radiation beam from the storage ring. This crystal and the second (L2) move and rotate in tandem to the appropriate positions to give a highly parallel, monochromatic beam of specified energy with a desired dimension.

**Figure 7:**
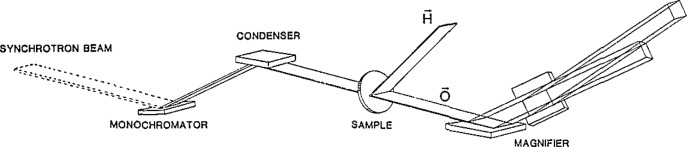
Image magnification: schematic beam path for two dimensional magnification. This example shows the image formation for the 0 (forward)-diffracted beam in transmission under the Bragg condition.

**Figure 8:**
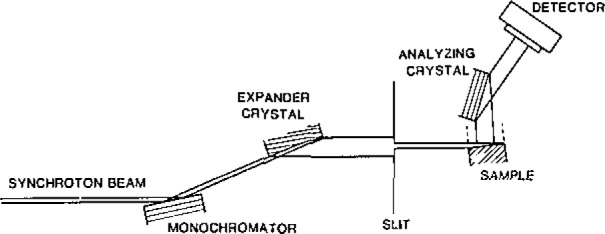
Schematic of experimental arrangement for “out state analysis.” A fourth crystal, in addition to two monochromator crystals and the sample being studied, passes the diffracted beam only over a narrow angular range, permitting observation either of defect images or of perfect (matrix) crystal images without interference from the other type of image.

It is important to have available monochromatic beams with variable dimensions and flux density. The monochromator crystals, L1 and L2, can be configured in three different ways: (a) symmetrical diffraction, resulting in magnification of one; (b) asymmetric diffraction in the magnification mode, (providing a large size, more highly parallel beam); and (c) asymmetric diffraction in the demagnification mode, [providing a concentrated, less parallel beam, useful for work with an x-ray magnifier [9] (fig. 7), as described later, and for “out state” analysis (fig. 8)].

Diffracted image magnifier crystals L3 and L4 in figure 6 can be placed orthogonally in the beam to produce two dimensional magnification by successive stages of asymmetric Bragg diffraction. Such an x-ray image magnifier produces an undistorted image, magnified up to 150-fold before detection either with film or with an image detector [9,10]. The submicron range of resolution in real time for crystals can thus be reached by commercially available two dimensional image detectors with a practical resolution of about 30 micrometers. Figure 7 illustrates the formation of a two dimensionally magnified image of a 0-diffracted (forward diffraction) beam in transmission. Such an x-ray magnifier can be used also as a zoom lens [6], since a given pair of two asymmetrically cut crystals provides continuously changing magnification as different energies of the incident beam are selected by the control system.

The use of flat crystal diffraction in an analyzer stage provides additional important opportunities for further analysis. Crystal L3 alone when set for asymmetric diffraction can act as an ideal angular analyser with an aperture less than one arc-second. This defines precisely the momentum transfer of x-ray photons detected after diffraction and scattering. In conjunction with an incident beam prepared to be extremely parallel by the monochromator system described above, such an analyzer crystal is capable of providing for equi-d mapping (lattice parameter), angle-resolved imperfection imaging, and small angle x-ray scattering (SAXS) imaging [11–15]. These analytic features strengthen diffraction imaging with a new quantitative capability in which the spatial imperfection information in diffraction images is preserved or enhanced. With these optical systems, microstructural effects that have been observed and measured *in situ* on a real time basis under simulated environmental conditions in white beam imaging now come within reach for monochromatic imaging.

## 4. Illustrative Examples

**Figure 9:**
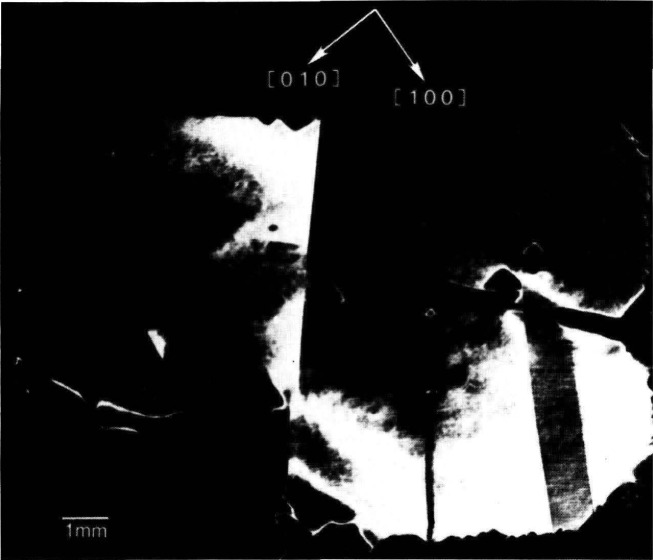
Image of 8 keV diffraction from (1 1 10) planes of a mosaic (001) cut mercuric iodide crystal.

A crystal containing distinct grains (which by definition differ in crystallographic orientation) yields broad or structured rocking curves in conventional x-ray diffraction. Diffraction images of such crystals typically display sharp contrast among the various grains. However, even where the difference in orientation is in excess of several arc-minutes, simultaneous diffraction from more than one grain can be observed and analyzed quantitatively if the crystal is suitably oriented with respect to the beam, as has been done in figure 9, where five grains can be distinguished. In such instances, the tilt of the individual grains and their orientation with respect to one another can be accurately determined from the clear displacement of the images of the various grains with respect to each other [16].

**Figure 10:**
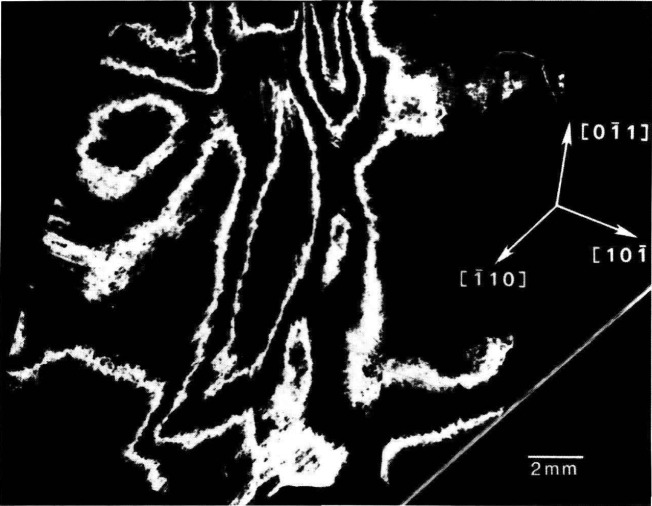
“Contour map” of strains in (111)-cut slice of cadmium telluride, recorded by superimposing diffraction from (333) planes of cadmium telluride recorded at three diffraction angles differing by 144 arc-seconds.

Broad rocking curves can arise also from a different source of imperfection, which, in the absence of images, is sometimes confused with the assumed presence of multiple grains or subgrains. One such case is shown in figure 10. The rocking curve for this crystal of cadmium telluride would approach in breadth the curve for the mercuric iodide crystal shown in figure 9. However, in contrast to the diffraction image of mercuric iodide, the image of cadmium telluride shows no major sharp boundaries separating crystal grains or subgrains. The breadth of the rocking curve in this instance is traceable to relatively gradual, irregular variation either in the lattice constant or in the lattice orientation. Improvement of such crystals will clearly depend on the particular types of crystal imperfection that are dominant. Steps taken to reduce the nucleation or propagation of additional grains will be ineffective in improving the quality of crystals whose perfection is limited principally by more subtle inhomogeneity within individual grains, perhaps due to impurities or deviation from stoichiometry.

**Figure 11:**
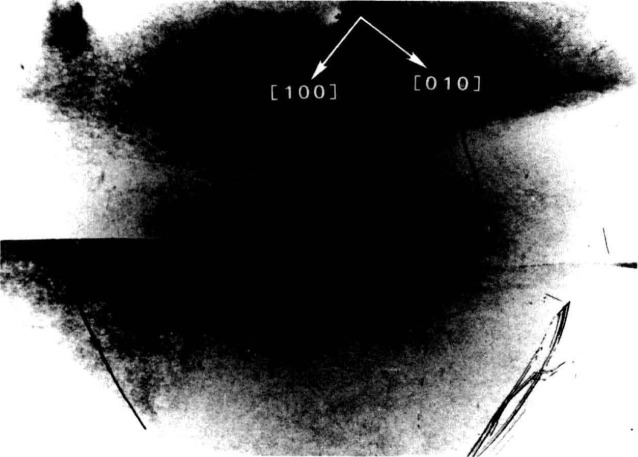
Sharp boundaries separating subgrains differing in orientation by about 2 arcseconds, shown in 8 keV diffraction from ( $\hat{2}\hat{2}4$) planes of a (001)-cut undoped gallium arsenide wafer. Also visible are curved regions of strain that produces curved, continuously varying contrast.

**Figure 12:**
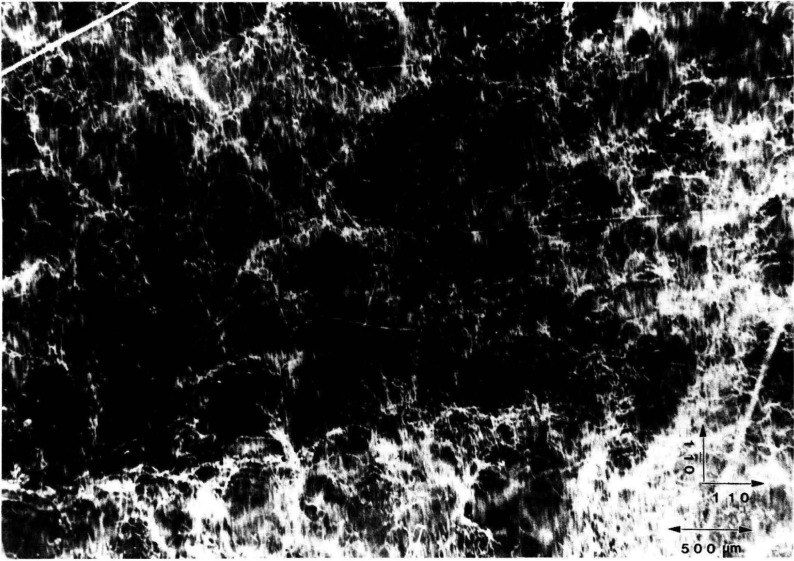
Boundary defects (possibly antiphase interfaces) observed in 10 keV diffraction in Laue geometry (transmission) from (220) planes of (001)-cut undoped gallium arsenide crystal.

Clearly delineated sharp subgrain boundaries are observed also in high quality crystals. Such boundaries can separate regions of high contrast even when the orientation of the subgrains differs only by a few arc-seconds in the direction of the beam, as in the case in figure 11. More complex boundaries can be resolved even under unfavorable conditions in which imperfections are more complex, as is characteristic of undoped gallium arsenide, shown in figure 12. The orientation of each feature is indicated by changes in the visibility with change in diffraction. Highly oriented features in some parts of this image result from boundary scattering in the interior of the crystal, while other features display sharp contrast characteristic of features located close to the surface.

**Figure 13:**
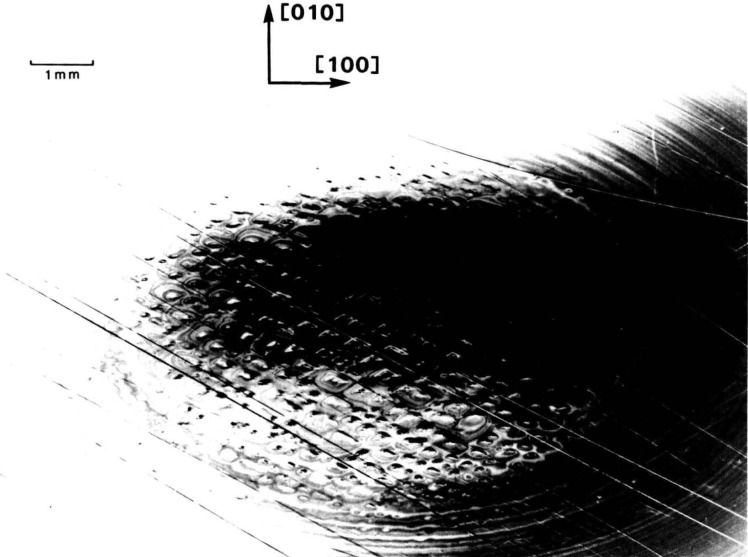
Image obtained in Bragg geometry diffraction at 8 keV from (004) crystal planes of the (001)-cut indium doped gallium arsenide wafer shown in figure 2 and 3 in Laue geometry.

**Figure 14:**
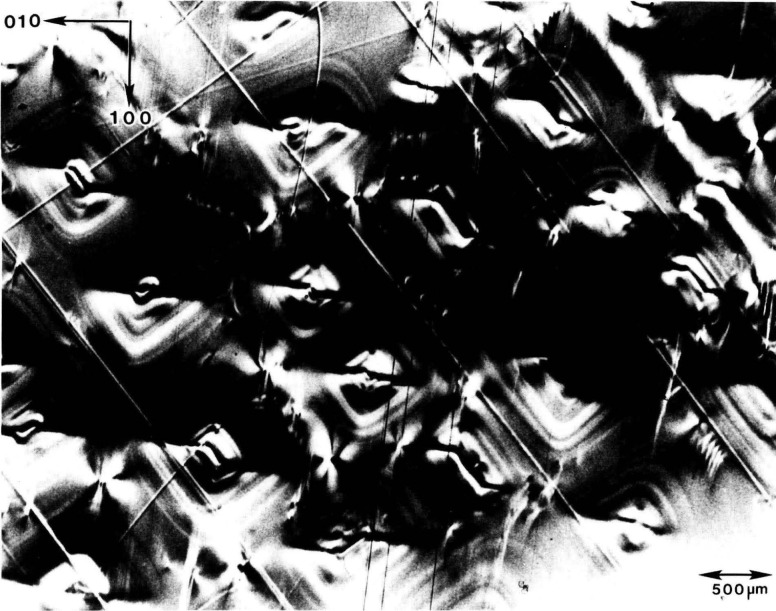
Magnified portion of image of diffraction in Laue geometry at 10 keV from (400) planes of the (001)-cut indium doped gallium arsenide crystal shown in figures 2, 3, and 13.

Other types of structure, clearly related to specific aspects of the growth of a crystal, are visible in indium doped gallium arsenide, as in figure 13. Geometrical features in a circular region of the center of a boule appear to imply faceted growth in this region. This is surrounded by striations associated with periodic fluctuation in one or more growth parameters. An image of the central region of this boule is shown magnified in figure 14. The rectangular features characteristic of this region are oriented along <110> directions. These different features in local regions indicate that the interface between the melt and solid is curved and changes in time during crystal growth [17].

## 5. Comprehensive Understanding

A variety of strains and other crystallographic irregularities can thus be portrayed by diffraction imaging. Through such observations, the role that these defects play can be correlated with specific aspects of materials properties [18]. However, also inherent in this analysis is the insight that it can provide into the origin of complex anomalies and the corresponding potential for their control where suitable samples are available for study.

**Figure 15:**
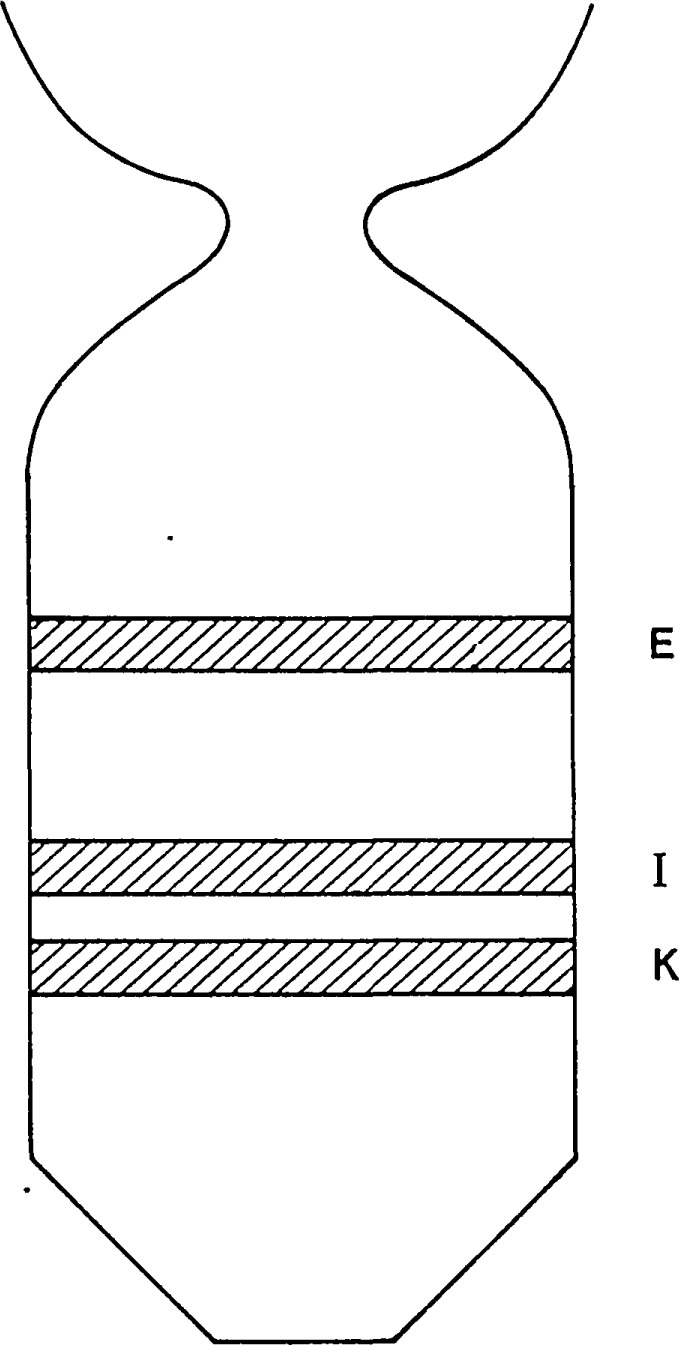
Relative positions of slices taken from a single high quality 8 mm boule of bismuth silicon oxide.

**Figure 16:**
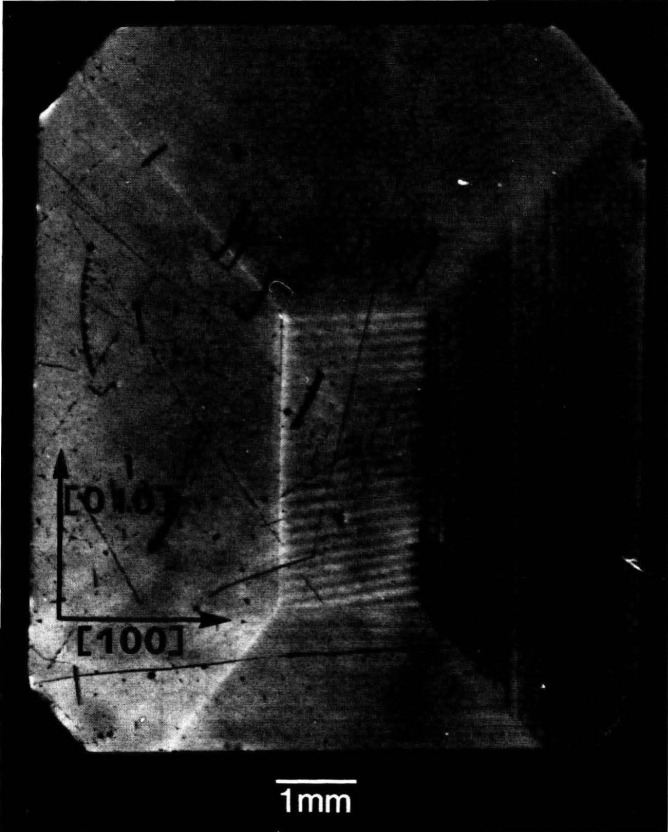
Diffraction image in Bragg geometry of (001)-cut slice E of bismuth silicon oxide from (006) planes at 8 keV.

**Figure 17:**
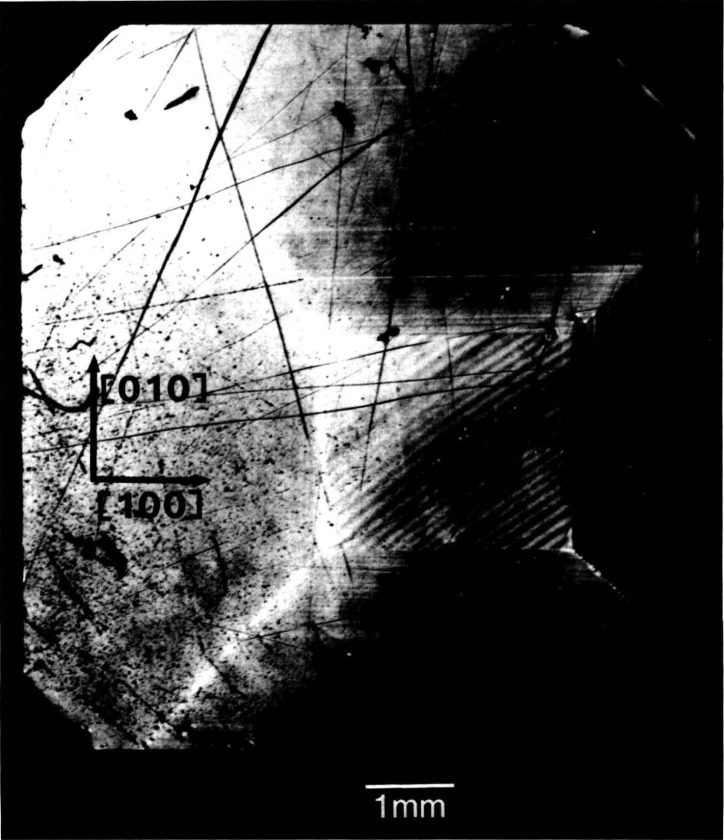
Diffraction image in Bragg geometry of (001)-cut slice I of bismuth silicon oxide from (0 0 10) planes at 8 keV.

**Figure 18:**
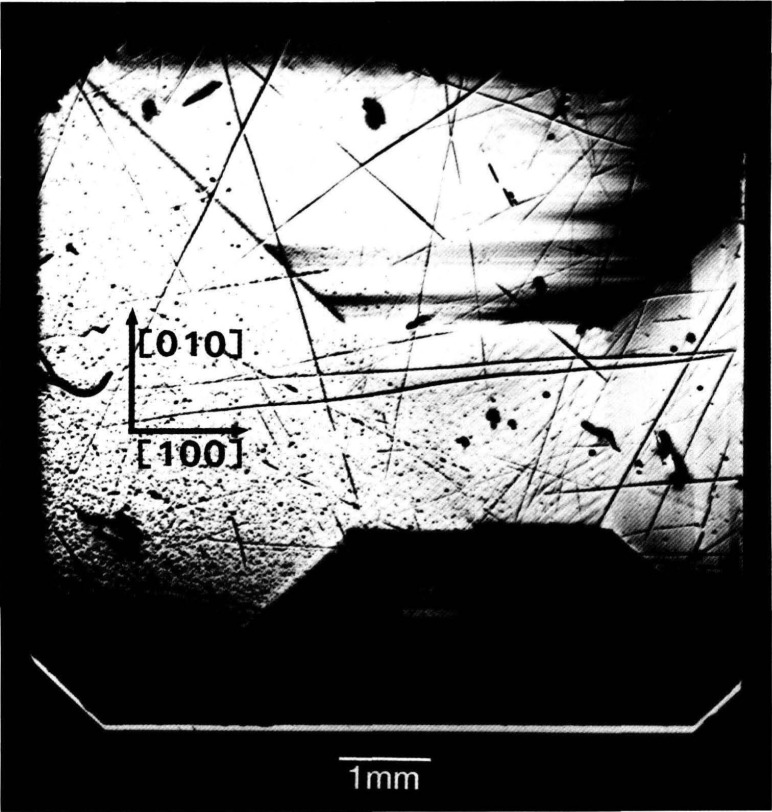
Diffraction image at 13.4 keV in Laue geometry from ( $0\bar{6}0$) planes of bismuth silicon oxide slice I, shown in Bragg geometry in figure 14.

**Figure 19:**
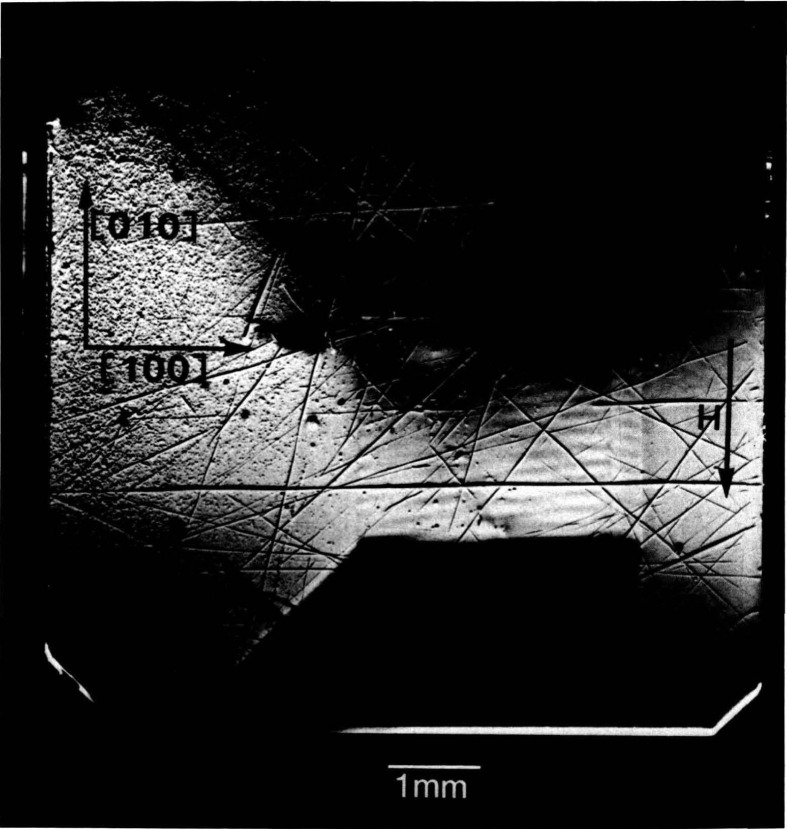
Diffraction image in Laue geometry of bismuth silicon oxide slice K from ( $0\bar{6}0$) planes at 13.4 keV. This crystal is shown in Bragg geometry in figure 1 and in another orientation in Laue geometry in figure 4.

An example of this aspect of monochromatic synchrotron diffraction imaging is a recent study carried out on three slices taken from a single high quality boule of bismuth silicon oxide grown in the [001] direction as shown in figure 15 [3]. The three slices were cut and polished perpendicular to the growth direction, the perimeter retaining the shape that it acquired during growth. Slice K is shown in Bragg (reflection) geometry in figure 1 and in Laue (transmission) geometry in one orientation in figure 4. Slices E and I are shown in Bragg geometry in figures 16 and 17, respectively. Slice I is shown in Laue geometry in figure 18. Slice K is shown in a second orientation in Laue geometry in figure 19.

**Figure 20:**
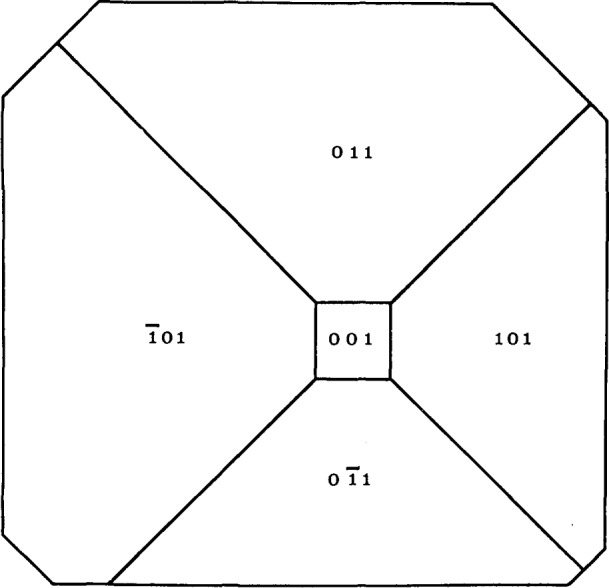
Abstraction of face of growing bismuth silicon oxide boule shortly after initial necking.

**Figure 21:**
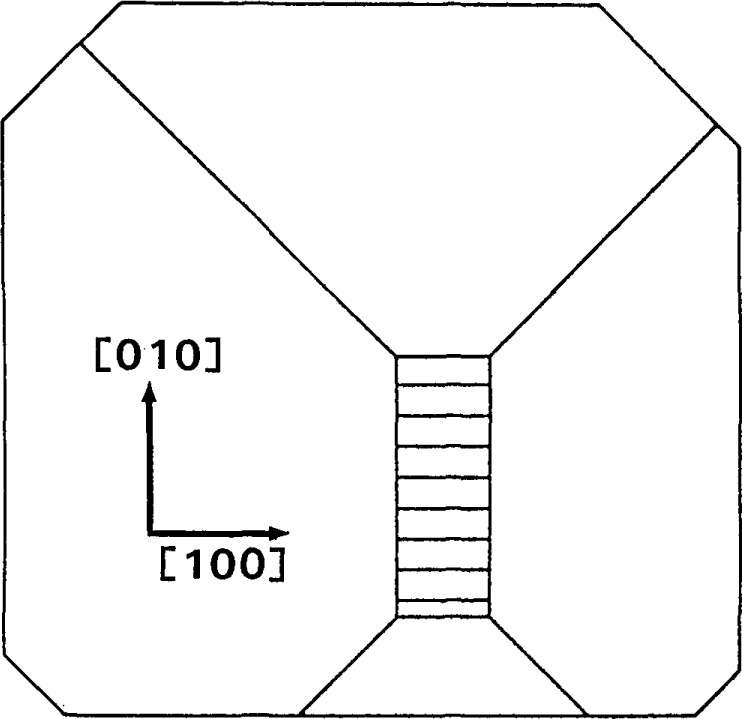
Abstraction of faceted growth of bismuth silicon oxide boule while slice E was being formed.

Analysis of these diffraction images permits us to unfold the following sequence of steps in the faceted growth of the boule. Shortly after necking, a small near-(001) growth plane had been established in the center of the growing face. Four peripheral {101} facets had also been formed. These are depicted in figure 20. By the time that the boule had grown to the location of slice E, the ( $0\bar{1}1$) facet was growing more rapidly than the others, causing it to decrease in area as illustrated in figure 21. Meanwhile, the central, near-(001) facet was growing nonuniformly. Its growth rate was greater in proximity to the rapidly growing ( $0\bar{1}1$) facet, causing the central near-(001) facet to deviate from a precise (001) orientation and resulting in strains observed as fringes in the central part of figure 16.

**Figure 22:**
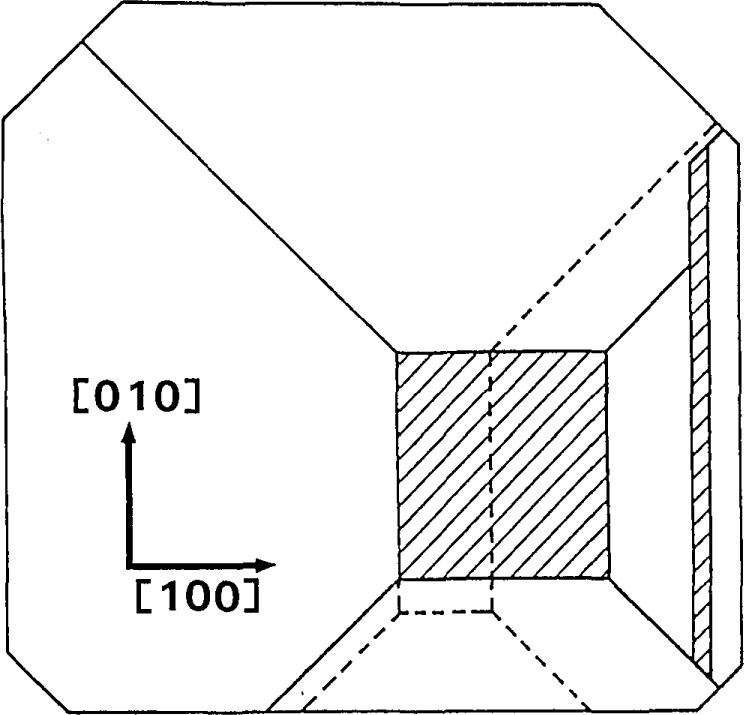
Abstraction of faceted growth of bismuth silicon oxide boule while slice I was being formed.

**Figure 23:**
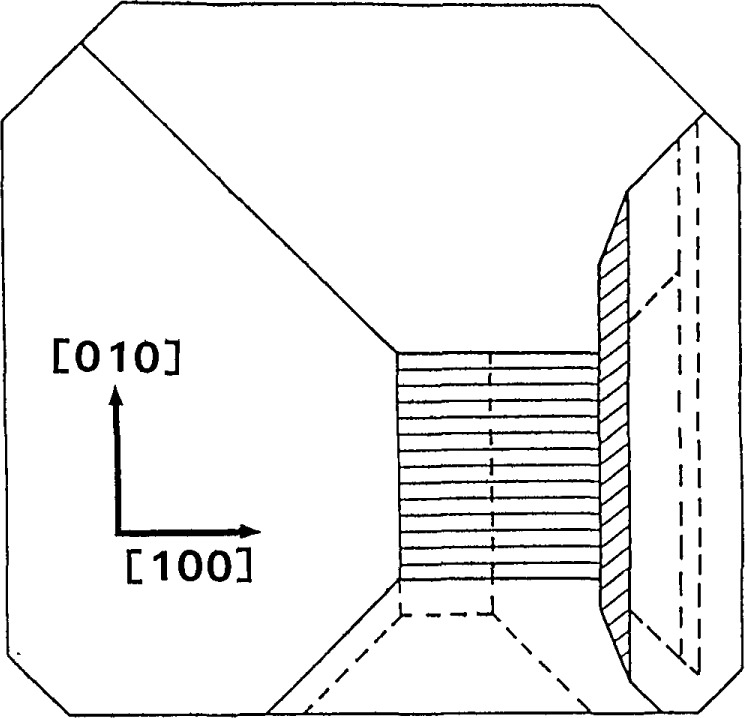
Abstraction of faceted growth of bismuth silicon oxide boule while slice K was being formed.

Between the growth of slice E and that of slice I, the rate of growth of the ( $0\bar{1}1$) facet slowed, while the growth of the (101) facet increased, causing a corresponding change in the shape of the related facets as abstracted in figure 22. The change in the orientation of the central figures indicates that the slope of the central near-(001) face underwent a corresponding change. By the time that the center of the boule had grown to position K, however, growth of the (101) facet had ceased completely, causing distinctive correlated high strain regions in both slices I and K, and associated further change in the orientation of the central strain fringes, figures 22 and 23, respectively.

**Figure 24:**
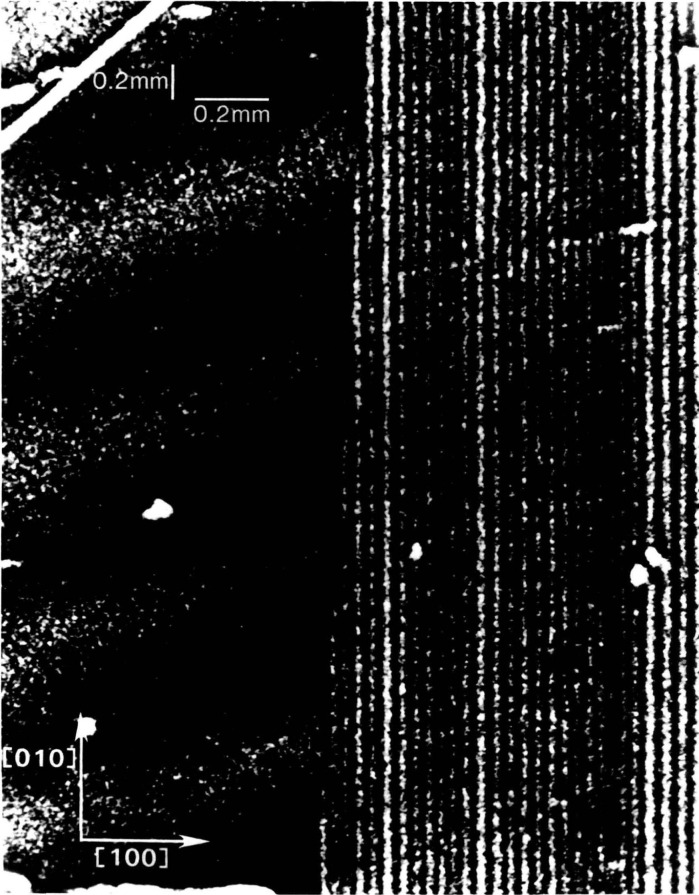
Magnification of region adjacent to the center, in the [100] direction, of a (006) diffraction of slice I in Bragg geometry at 8 keV. This section shows the boundary between the central rectangular near (001) facet growth region containing fringes and the peripheral (101) facet growth.

**Figure 25:**
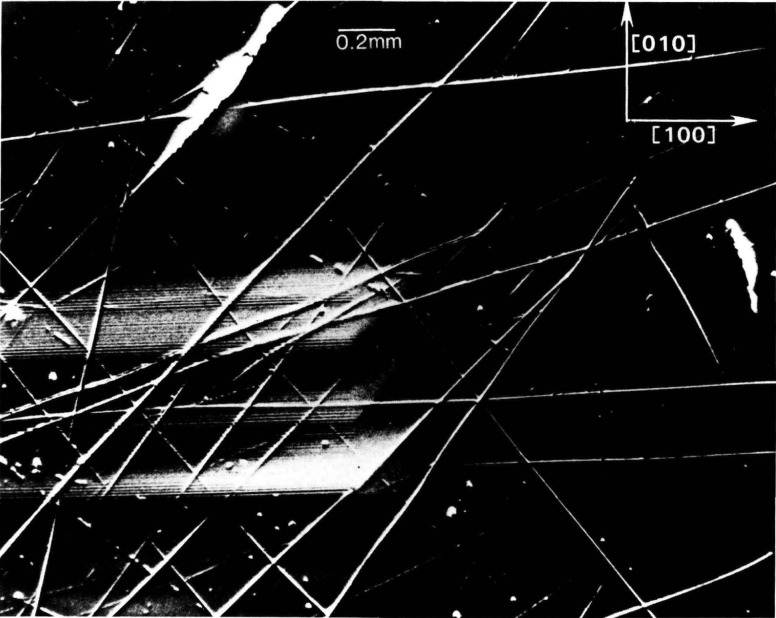
Magnification of the [110] comer region of a ( $0\bar{6}0$) diffraction image of slice K in Laue geometry at 13.4 keV. This image shows strains in the [010] direction, and thereby the cessation in the growth of one of the facets while an adjacent facet continued to grow.

Specific aspects of this nonconstant faceted growth model are verified in magnified portions of the diffraction images, figures 24 and 25. Figure 24 illustrates continuity between fringes observed in the central region and the peripheral striations, supporting the faceted growth model where growth of the central facet is at a slight angle to the (001) plane. Cessation in the growth of the (101) facet while the (001) facet continued to grow is illustrated in figure 25.

**Figure 26:**
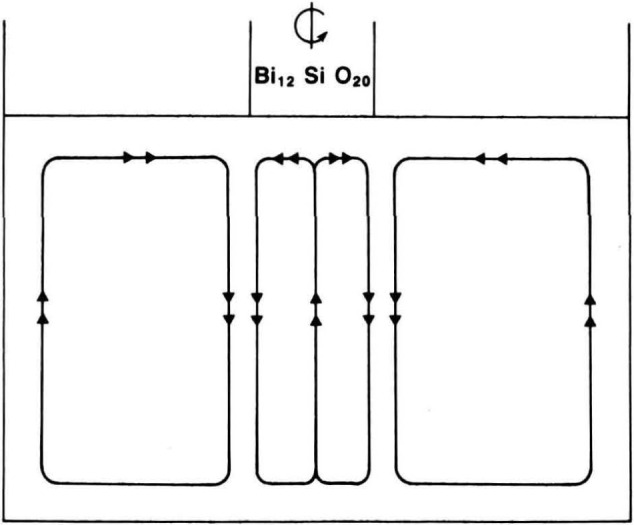
Schematic illustration of basic elements in a model for the flow in the bismuth silicon oxide melt as modified for stable flat interface Czochralski growth.

**Figure 27:**
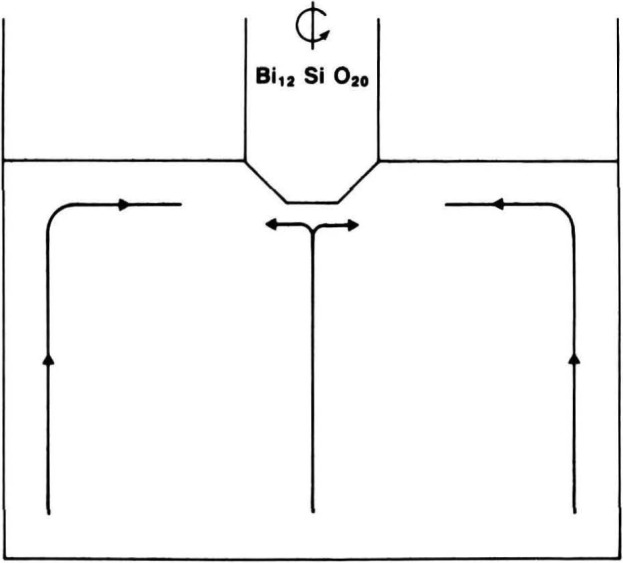
Schematic illustration of basic elements in the model for the flow in the bismuth silicon oxide melt as modified for faceted interface Czochralski growth. Competition between the two melt currents causes changes in the relative rates of growth of the various facets.

The principal elements of a mathematical model that successfully predicts flow during stable Czochralski growth for a flat interface [19] are illustrated schematically in figure 26. An outer flow cell is established by convective heating of the crucible, while an opposing inner flow cell is established by rotation of the growing boule. The general features of this mathematical model have been verified experimentally [19]. However, the asymmetrical faceted growth found in the diffraction images requires a modification in this model. The development of facets influences, and in turn is influenced by, changes in the position of the boundary between the two flow cells, indicated in figure 27. The changes in flow are associated with corresponding changes in the temperature of particular growing regions of the boule and hence control the growth morphology.

## 6. Devices

**Figure 28:**
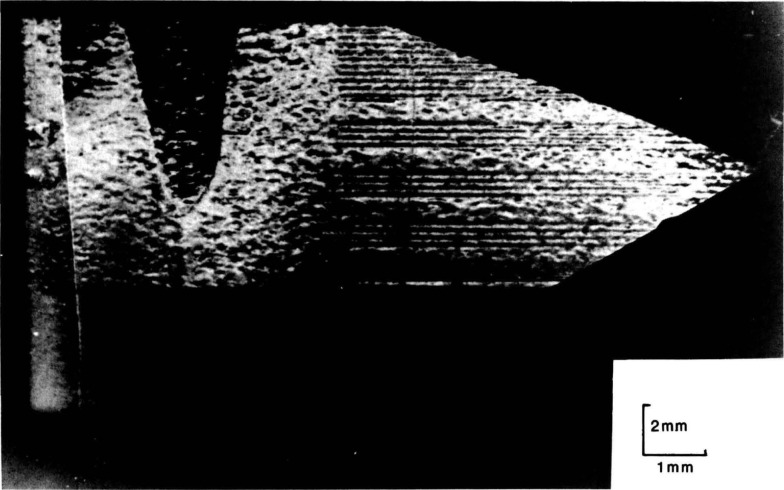
Diffraction image of titanium indiffused lithium niobate showing strains caused by this stage in the manufacture of a guided wave device.

Modification in materials that can be observed after an additional stage of processing in the manufacture of a device is illustrated in figure 28. This image shows strains in a piece of lithium niobate indiffused with titanium. Such regions of the crystal will guide light to be switched or modulated by electrodes that have yet to be installed. Observations can be made first on a virgin material and at various stages in the finishing of the crystal and subsequent processing of a device in order to identify the stages at which critical defects are introduced. Another example is the observation of EL2 defects, which control the electrical compensation of semi-insulating materials such as gallium arsenide, and their correlation by laboratory topography with dislocation density [18].

## 7. Future Directions

**Figure 29:**
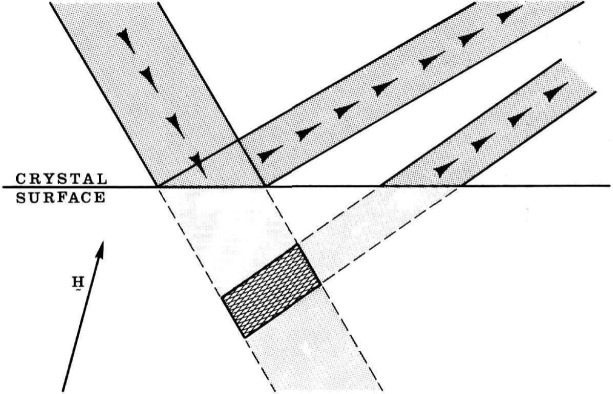
Schematic diagram of sources of image confusion caused by the unfortunate combination of defect strain and lattice orientation.

**Figure 30:**
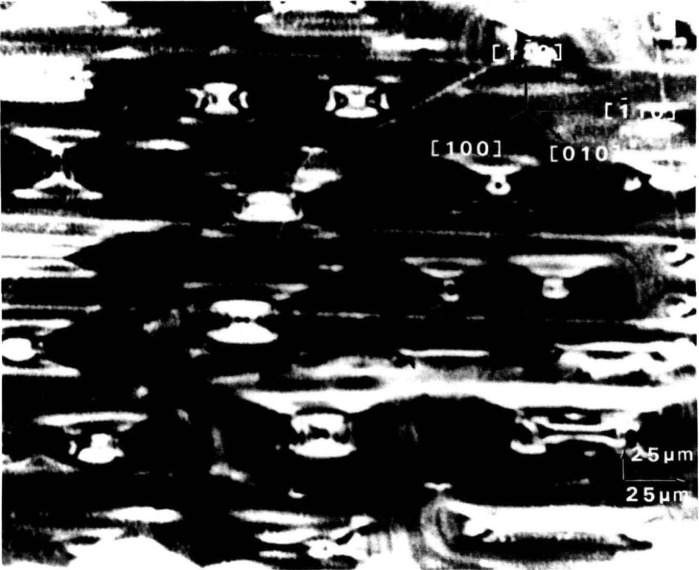
Magnified portion of (004) diffraction image of central portion of (001)-cut indium doped gallium arsenide wafer as diffracted by a silicon crystal oriented to pass the “perfect crystal” diffraction and to discriminate against defects.

**Figure 31:**
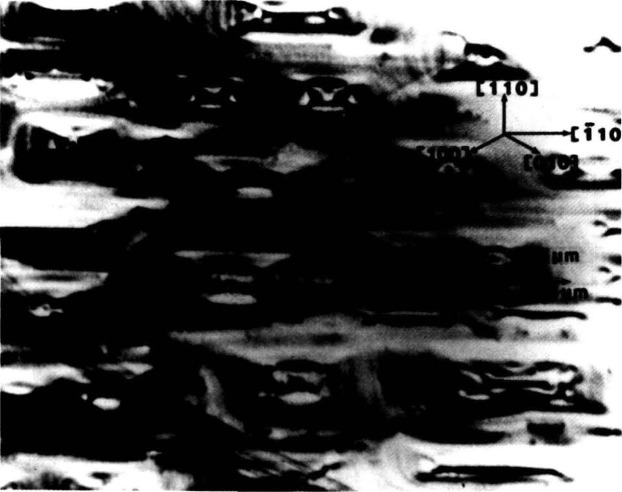
Magnified portion of (004) diffraction image of central portion of (001)-cut indium doped gallium arsenide wafer as diffracted by a silicon crystal oriented to pass diffraction from defects and to discriminate against “perfect crystal” diffraction.

While imaging with monochromatic radiation removes ambiguity associated with diffraction of white radiation having a range of wavelengths, some ambiguity in the interpretation nevertheless remains. Contrast in a monochromatic image can be caused either by lattice parameter variations or by variation in orientation of the crystal lattice. Moreover, because of differing diffraction (scattering) angles, radiation from a defect appears in an image displaced from the image of the immediately surrounding undisturbed region. Diffraction from a surface defect surrounded by a perfect crystal is illustrated schematically in figure 29, in which spatial confusion in the diffracted image is shown and is clearly traceable to dissimilar diffraction (scattering) angles. Separation of the diffracted radiation from the two sources, defect and surrounding region, and removal of the ambiguity in the source can be accomplished by “out state analysis” through placing an additional analyzing crystal so as to intercept the diffracted image, as illustrated in figure 8, where an analyzing crystal is depicted in “symmetrical diffraction.” Examples of the results of such “out state analysis” are shown in figures 30 and 31, which picture images of the central region of the indium doped gallium arsenide crystal previously shown in conventional diffraction images in figures 2, 3, and 13.

When the analyzer crystal is set to pass “perfect crystal” diffraction, the contrast between diffracting and non diffracting regions is relatively high. This high contrast is caused by the interaction between the beam in perfect crystal diffraction and the beams scattered from defects. When the analyzer crystal is set to discriminate against this “perfect crystal” diffraction from the matrix, the image is then restricted to the scattering from the defects. Analysis of such simplified images should prove to be more direct and thus less subject to models of defect location than is the analysis of conventional diffraction images.

Another opportunity with monochromatic synchrotron radiation not yet fully exploited is the ability to carry out experiments in real time while observations are made with a video camera. One example whose feasibility has been proved is the observation of changes in the strain in electrooptic crystals as various electromagnetic fields are applied. Strains have been observed in real time in lithium niobate as electrostatic fields are applied [20]. These strains are highly nonuniform, relax with time, and display hysteresis.

In summary, diffraction imaging can provide a great deal of insight into the physics and formation of electrooptic, photorefractive, and gamma ray sensitive crystals. Through exploitation of these opportunities, we expect to expand our understanding of the role of defects, which may either reduce or enhance the physical properties of these materials. Through collaboration with crystal growers, we can thus optimize these parameters through increase in the incidence and character of those defects that can be utilized and reduction of those that interfere.
